# OP32 Identification Of Factors Alongside Costs And Effectiveness For The Technology Assessment Of Comprehensive Genomic Profiling: A Systematic Review

**DOI:** 10.1017/S0266462324000941

**Published:** 2025-01-07

**Authors:** Lucas van Schaik, Ellen Engelhardt, Erica Wilthagen, Neeltje Steeghs, Andrea Fernandez Coves, Manuela Joore, Wim van Harten, Valesca Retèl

## Abstract

**Introduction:**

Comprehensive genomic profiling (CGP) identifies many targets at once. However, it is challenging for reimbursement decision-makers to incorporate all potential effects in their assessment. The aim of this study is twofold: first, to identify which factors, besides effectiveness and costs, might influence the choice for CGP in advanced cancer patients, and second, to identify the available evidence for these factors.

**Methods:**

We performed a systematic literature review in MEDLINE, Embase, and Scopus with a two-step design. First, a scoping search was performed to identify relevant factors. Extracted factors were grouped with domains of the EUnetHTA core model and ISPOR (Professional Society for Health Economics and Outcomes Research) “value flower.” Two expert sessions were held to validate factors and construct definitions. Second, a systematic search was conducted to identify the available empirical evidence for these factors. Eligibility criteria for the systematic search were the use of CGP (≥200 genes), advanced cancer patients, and the presentation of empirical evidence on one of the factors.

**Results:**

Five factors were identified in the scoping search: “feasibility” (adopting tests in the health care system), “test journey” (pathway from requesting tests until reporting of results), “wider implications of diagnostic results” (impact of test beyond identifying on-label treatments), “organization of laboratories” (organization of tests and access to tests), and “scientific spillover” (learnings of testing). Eighty-three articles were included following the systematic search, and empirical evidence was identified for the factors “test journey” and “wider implications of diagnostic results”. Few studies had adequate comparative study designs. Heterogeneity was observed among studies in the definitions of outcomes and the reported evidence.

**Conclusions:**

Comprehensive reimbursement decision-making for CGP can be supported by including the five identified factors. However, quantifiable evidence was only identified for the “patient test journey” and “wider implications of diagnostic results”. Current literature provides limited high-quality evidence to establish the added benefit of CGP, as adequately designed comparisons are lacking. For evidence-based decision-making, uniform outcome measurements are recommended.

